# Molecular Proof of a Clinical Concept: Expression of Estrogen Alpha-, Beta-Receptors and G Protein-Coupled Estrogen Receptor 1 (GPER) in Histologically Assessed Common Nevi, Dysplastic Nevi and Melanomas

**DOI:** 10.3390/medicina57111228

**Published:** 2021-11-11

**Authors:** Magdalena Spałkowska, Grzegorz Dyduch, Elżbieta Broniatowska, Giovanni Damiani, Anna Wojas-Pelc

**Affiliations:** 1 Department of Dermatology, Faculty of Medicine, Jagiellonian University Medical College, 31-501 Krakow, Poland; magdalena.spalkowska@uj.edu.pl (M.S.); awpklinika@gmail.com (A.W.-P.); 2 Department of Rheumatology and Immunology, University Hospital in Krakow, 30-688 Krakow, Poland; 3 Department of Pathology, Faculty of Medicine, Jagiellonian University Medical College, 33-332 Krakow, Poland; grzegorz.dyduch@uj.edu.pl; 4 Faculty of Medicine and Health Sciences, Andrzej Frycz Modrzewski Krakow University, 30-705 Krakow, Poland; ebroniatowska@afm.edu.pl; 5 Clinical Dermatology, IRCCS Istituto Ortopedico Galeazzi 4, 20161 Milan, Italy; 6 Department of Biomedical, Surgical and Dental Sciences, University of Milan, 20122 Milan, Italy; 7 PhD Degree Program in Pharmacological Sciences, Department of Pharmaceutical and Pharmacological Sciences, University of Padua, 35131 Padua, Italy

**Keywords:** melanoma, dysplastic nevus, estrogen, GPER, estrogen receptor, gender, precision medicine

## Abstract

*Background and Objectives:* Epidemiologic data show significant differences in melanoma incidence and outcomes between sexes. The role of hormonal receptors in the pathogenesis of melanocytic lesions remains unclear, thus we performed this study aiming to assess estrogen receptors expression in different melanocytic lesions. *Materials and Methods:* We performed a cross-sectional study that included 73 consecutively excised melanocytic lesions. Estrogen receptor alpha (ERα), beta (ERβ), and G-protein coupled estrogen receptor (GPER) expression was analyzed in melanocytes and keratinocytes of common nevi, dysplastic nevi, melanoma, healthy skin margin, and in sebaceous and sweat gland cells. *Results:* ERβ expression was higher in dysplastic nevi margin melanocytes compared to common nevi (*p* = 0.046) and in dysplastic nevi keratinocytes compared to melanoma keratinocytes (*p* = 0.021). ERβ expression was significantly higher in margin melanocytes compared to melanoma melanocytes (*p* = 0.009). No difference in ERβ expression was shown between melanocytes of three types of lesions. GPER expression was higher in nuclei and cytoplasm of dysplastic nevi (*p* = 0.02 and *p* = 0.036 respectively) and at the margin compared to melanoma. GPER expression was lower in sebaceous glands of tissue surrounding common nevi (*p* = 0.025) compared to dysplastic nevi. GPER expression was higher in skin margin tissue melanocytes (*p* = 0.016 nuclear, *p* = 0.029 cytoplasmic) compared to melanoma melanocytes. There were no differences in ERα expression between the melanocytic lesions. *Conclusion:* Further large-scale studies are warranted to investigate the potential role of ERβ and GPER in the pathogenesis of melanocytic lesions.

## 1. Introduction

Estrogen receptors (EA) are three receptors (ERα, ERβ and G-protein coupled estrogen receptor (GPER)) belonging to the nuclear steroid hormone receptor superfamily (ERα, ERβ) and to the G protein-coupled receptor superfamily. Their subtypes prevalence is species-specific, tissue- and cell-specific due to their multiple activities in mediating 17β-estradiol (E2) functions in eukaryotes [1].

ERα, also known as NR3A1, is a 595 amino acid protein, derived from the Estrogen Receptor 1 (*ESR1)* gene on the 6q25.1 chromosome region, with three main isoforms and three functional domains (DNA-binding domain, ligand-binding domain, N-terminal domain). ERβ, also known as NR3A2, is a 530 amino acid protein transcripted and traduced from *ESR2* gene in the 14q23.2 chromosome region with a high structural homology with ERα and 5 isoforms [2]. GPER, also known as GPR30, is the last discovered in the EA. This 375 amino acid membrane-based protein has 7 transmembrane a-helical regions, 4 extracellular and 4 cytosolic segments derived from *GPER1* gene located in the 7p22.3 chromosome region [3].

EA coordinate several physiological functions included and not limited to glucidic homeostasis [4] and even aging [5]. Interestingly their deregulation was recently linked to several pathological conditions spacing from cancer (i.e., breast [6], ovarian [7] and cholangiocarcinoma) to joint and muscle disorders [8,9,10]. Conversely, in melanoma the EA modulation remains highly debated also if epidemiological and clinical data may suggest a possible involvement. Melanoma incidence is higher in men compared to women, and men tend to have poorer outcomes [11,12,13]. Furthermore, melanoma incidence starts rising after puberty, and among women starts to decrease after the 5th decade of life (which corresponds to menopausal age), while in men it remains steady, resulting in nearly three times higher incidence for men aged 75 and over compared to their female counterparts [14,15,16,17]. Male gender also significantly impacts outcomes and related costs [18], with males having a greater risk of positive sentinel lymph node, metastasis and a lower melanoma specific survival compared to females [19,20]. In addition, there are differences in immunotherapy response between men and women [21] as well as melanoma location [22].

These differences may be due to estrogens, which stimulate melanocytes differentiation and melanin production, by estrogen receptors alpha (ERα) which is a gene transcription activator and is involved in neoplasm progression, and beta (ERβ), which is antagonistic to ERα [23,24,25,26,27]. Prior to menopause, there is a higher proportion of ERβ to ERα in the skin, and after menopause this proportion starts to decrease, mostly due to decrease in ERβ [25,28]. This is supported by the finding that women of childbearing age with advanced melanoma had higher survival rates than post-menopausal women [29,30,31,32].

Since EA deregulation is present in several cancer types we expect a statistically significant expression of ERα, ERβ and GPER in common nevi, dysplastic nevi and melanoma due to their different biological behavior.

## 2. Materials and Methods

### 2.1. Study Population

In this cross-sectional study we enrolled 73 consecutive patients referred to the Department of Dermatology, Jagiellonian University Medical College in Krakow, qualified to surgical excision of melanocytic lesion based on medical history, clinical and dermatoscopic examination. In all cases an excisional biopsy of the lesions was performed according to standard procedures. All patients underwent a hormonal evaluation with quantification of gonadotropins (LH and FSH), total testosterone, prolactin, dehydroepiandrosterone and thyroid-stimulating hormone.

Fresh skin samples were subsequently placed in 10% neutral buffered formalin for fixation and transferred to Department of Pathology, Jagiellonian University Medical College in Krakow for further histopathological analysis.

We analyzed the expression of ERα, ERβ, GPER and progesterone receptors in melanocytes and keratinocytes of melanoma, melanocytic nevi and dysplastic nevi, in melanocytes and keratinocytes of healthy skin margin, and in sebaceous and sweat gland cells. Standard histological procedures were used for tissue preparation.

The study was approved by local bioethical committee (No 122.6120.62.2015, 30 April 2015 Komisja Bioetyczna Uniwersytetu Jagiellońskiego).

### 2.2. Inclusion and Exclusion Criteria

We included patients that signed the informed consent form to remove and analyze a pigmented lesion with a final diagnosis of melanoma, melanocytic nevi and dysplastic nevi. Remarkably, we excluded patients (i) with a previous neoplasia (<5 years) affecting the reproductive system, (ii) undergoing treatments with primary or secondary effects on sexual hormones (i.e., retinoids), (iii) with a recent chemo/radiotherapy (<5 years), (iv) with recent spontaneous or induced abortions (<2 years).

Oral contraceptives use was not an exclusion criterion.

### 2.3. Immunohistochemistry

Formalin-fixed paraffin-embedded tissue was cut into 3 μm sections and mounted onto the silanized slides. Tissue slides were deparaffined in xylene (in temperature 60 °C, three changes-duration: 10 min, 15 min, 15 min) and hydrated (in room temperature, three changes-5 min each). Sections were incubated in room temperature for 10 min in 3% H_2_O_2_ solution in order to inhibit endogenous peroxidase. Samples were subsequently washed in distilled water and incubated with TBS buffer for 5 min. Heat-induced epitope retrieval was performed by heating slides in 97 C. After unmasking the samples *Ultra Vision Protein* Block (ThermoScientific, Cheshire, UK) was applied for 5 min in room temperature in humidity chamber. For detecting target proteins, the following primary antibodies were applied and incubated in room temperature in humidity chamber: anti-estrogen receptor alpha monoclonal mouse antibody 6F11 Novocastra, (Leica Biosystems, Newcastle upon Tyne, UK) (dilution 1:100, incubation 30 min); anti-estrogen receptor beta mouse monoclonal antibody NR3A2 (Novus Biologics, Littleton, CO, USA) (dilution 1:50, incubation 30 min); anti-GPER GPR30, rabbit monoclonal antibody NLS1183 (Novus Biologics, Littleton, CO, USA) (dilution 1:50, incubation 30 min), anti-progesterone receptor mouse monoclonal antibody PgR636 (Dako Cytomation, Carpinteria, CA, USA) (dilution 1:100, incubation 60 min). The excess of antibody was washed with TBS buffer and post antibody blocking for Bright Vision plus (BrightVision+ Goat anti-mouse/rabbit HRP, ImmunoLogic, Duiven, The Netherlands) was applied for 20 min, then samples were incubated in moist chamber in room temperature for 30 min. The excess of reagent was washed with TBS buffer and poly-HRP–GAMs/Rb IgG (BrightVision+ Goat anti-mouse/rabbit HRP, ImmunoLogic, Duiven, The Netherlands) was applied, then incubated in room temperature, in moist chamber for 30 min. The excess of reagent was washed with TBS buffer and DAB Quanto (ThermoScientific, Cheshire, UK) was applied, then incubated in room temperature, in moist chamber for 3–8 min. The excess of reagent was removed by distilled water and stained with hematoxylin (ThermoScientific, Cheshire, UK). Samples were dehydrated by three alcohol and xylene changes in increasing concentrations and covered with cover glasses (CYTOSEAL, ThermoScientific, Cheshire, UK).

### 2.4. Assessment of Immunostaining

The expression of each receptor was measured using immunohistochemistry and expressed as a percentage of the cells stained (nuclei and additionally cytoplasm in case of GPER in 1 mm^2^). The expression was regarded as negative in cases where less than 5% of cells were stained. The result was assessed by two independent observers (G.D. and E.B.) and expressed as medium value.

### 2.5. Statistical Analysis

Due to relatively small number of analyzed specimens, and lack of normal distribution, Mann-Whitney U test was used to examine the impact of dichotomous variables on receptor expression. To compare the expression of each receptor between three groups of melanocytic lesions we used Kruskal-Wallis one-way analysis of variance by ranks. Post-hoc analysis was performed in case of significant results of Kruskal-Wallis test. Wilcoxon test was used to compare the expression of receptors between melanocytic lesion and healthy skin tissue margin. *p*-value below 0.05 was considered statistically significant for two-tailed tests. All the calculations were performed by the means of *STATISTICA* version 12 (StatSoft Inc., Tulsa, OK, USA).

## 3. Results

The studied group included 48 women and 25 men, the median age was 43 years (interquartile age—IQR 32–58). The studied group included patients with the following phototypes according to the Fitzpatrick scale: 35.62% with phototype I, 56.16% with phototype II and 8.22% with phototype III. 11% of patients had positive family history of melanoma, 47.95% positive history of sunburns and 34.25% history of sunbed use. The most common comorbidity was hypertension and it was present in 26.03% of patients. Interestingly, 23.29% of studied group had dysmenorrhea, 4 women (8.33%) presented with polycystic ovary syndrome, 8 women (16.67%) underwent oral contraceptive pills treatment during the study.

After pathological analyses, 25 excised lesions were diagnosed as common nevi, 24 as dysplastic nevi and 24 as melanomas.

There was a positive correlation between expression of ERβ in melanocytes of melanocytic lesions and patients’ age (*p* = 0.035, R = 0.25), whereas expression of cytoplasmic expression of GPER in melanocytes of healthy tissue margin was correlated negatively with patients’ age (*p* = 0.022, R = −0.27). The expression of other receptors did not correlate with patients’ age. There were no significant differences in estrogen α, β and GPER expression in melanocytes, keratinocytes of melanocytic lesions and margins, as well as glands between women in childbearing age, post-menopausal women and men.

**Figure 1:**
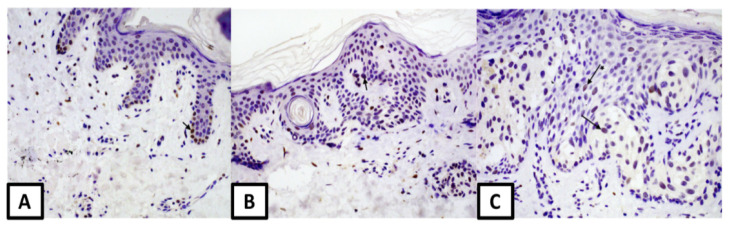
Nuclear expression of estrogen receptor α: (**A**) in melanocytic nevus (magnification 100×), arrow—melanocyte in the basal layer (**B**) in dysplastic nevus (magnification 100×), arrow—melanocyte (**C**) in melanoma (magnification 200×), arrows: melanocyte and * keratinocyte.

We found no differences in ERα expression in keratinocytes, melanocytes, sweat glands, and sebaceous glands between common nevi, dysplastic nevi and melanomas (Table 1 and Table 2) (Figure 1).

**Figure 2:**
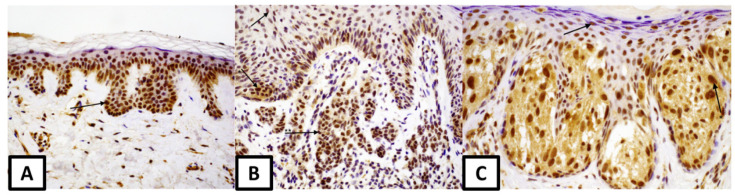
Nuclear expression of estrogen receptor β: (**A**) in melanocytic nevus (magnification 100×), arrow—melanocyte, (**B**) in dysplastic nevus (magnification 200×), arrows: keratinocyte and *, ** melanocytes (**C**) in melanoma (magnification 200×), arrows—keratinocyte and * melanocyte.

There was a difference in ERβ expression in margin melanocytes between common nevi and dysplastic nevi and it was higher at the margins of dysplastic nevi (median 90% vs. 100%, *p* = 0.05) (Figure 2).

Melanoma keratinocytes had lower expression of ERβ compared to dysplastic nevi (median 80% vs. 90%, *p* = 0.02). No difference in ERβ expression was shown between melanocytes of three types of melanocytic lesions.

**Figure 3:**
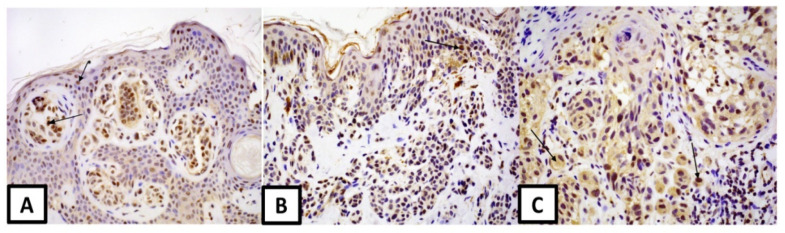
Expression of GPER receptor: (**A**) in melanocytic nevus (magnification 200×), arrows: melanocyte and * keratinocyte, (**B**) in dysplastic nevus (magnification 200×), arrow—melanocyte, (**C**) in melanoma-nuclear and cytoplasmic expression is observed (magnification 400×)-arrows showing cytoplasmic and nuclear * GPER expression.

GPER expression was higher in nuclei and cytoplasm of dysplastic nevi (respectively: median 45% vs. 25%, *p* = 0.02; median 70% vs. 30%, *p* = 0.04) and at the margin compared to melanoma (Figure 3).

In addition, GPER expression was lower in the sweat and the sebaceous glands of tissue surrounding common nevi (median 50% vs. 70%, *p* = 0.03, median 50% vs. 70%, *p* = 0.03) compared to dysplastic nevi.

We detected no difference in expression of ERα between melanocytes of melanomas and melanocytes of healthy skin margins, as well as between keratinocytes of melanomas and keratinocytes of healthy skin margins (Table 3).

ERβ expression was significantly higher in margin melanocytes compared to melanocytes of melanoma (90% vs. 85%, *p* = 0.01). No difference in expression of ERβ between keratinocytes of melanomas and keratinocytes of healthy skin margin was found. GPER expression was significantly higher in skin margin tissue melanocytes (80% vs. 25%, *p* = 0.02 nuclear, 80% vs. 30%, *p* = 0.03 cytoplasmic) compared to melanoma melanocytes. Keratinocytes of melanoma margin and melanoma tissue did not differ in GPER expression.

## 4. Discussion

In this study, for the first time, we compare the expression of three types of estrogen receptors between common nevi, dysplastic nevi and melanomas.

Estrogen receptors α and β are nuclear transcription factors with a variety of physiologic roles. ERα is the main estrogen receptor in the skin, however, despite this, increased levels of the receptor do not appear to play a role in malignant transformation of cutaneous lesions or melanoma pathophysiology [33]. This is in accordance with our findings of no differences in ERα expression in keratinocytes, melanocytes, sweat glands, and sebaceous glands between common nevi, dysplastic nevi and melanomas. Furthermore, in a sample of 38 melanomas, estrogen α receptor expression was negative [34]. In contrast, Schmidt et al. analyzed 94 melanocytic lesions and found that the majority of melanomas expressed Erα [35].

In contrast to ERα, ERβ plays a significant role in antitumor activity, as demonstrated by our finding of lowest ERβ expression in melanomas and dysplastic nevi and common nevi. In addition, we found that no difference in ERβ expression was shown between melanocytes of three types of melanocytic lesions. In a study of 94 melanocytic lesions, all expressed ERβ, with dysplastic nevi with high cytological atypia and lentigo maligna having the highest expression [35]. Similarly, among 60 melanoma lesions, all expressed ERβ as the dominant receptor [36].

De Giorgi et al. showed the expression of estrogen receptor β in 66 cases of superficial spreading melanomas. Hair follicles, sweat and sebaceous glands were characterized by high expression estrogen receptor β. Expression of estrogen receptor β was significantly lower in melanoma tissues than in healthy skin margin (median 50% vs. 80%, *p* < 0.0001) [37]. In our study we also showed significant differences in estrogen β expression between healthy skin margin and melanoma tissue. Expression of estrogen receptor β is in our study was higher in healthy margin melanocytes than in melanoma melanocytes. In the study by de Giorgi et al. the expression of estrogen receptor β in melanoma and healthy tissue margin was significantly lower in men than in women [37]. Authors showed higher level of expression of estrogen beta receptor in patients with BMI > 25 than in patients with BMI < 25 kg (80% vs. 70%, *p* = 0.04, result was insignificant after adjustment to age). Study showed that expression of estrogen receptor β was lower in post-menopausal women than in pre-menopausal women (50% vs. 70%, without statistical significance) [37]. In our study we did not show the correlation between BMI and estrogen β expression. In our study we did not show the differences in expression of estrogen β receptor between women in childbearing age and post-menopausal women. In our study, similarly to Giorgi et al. the expression of estrogen β receptor was higher in healthy tissue margin than in melanoma tissue. We showed correlation between expression of estrogen β receptor in melanocytic lesions and age of the patients (*p* = 0.035, R = 0.25), the correlation was weak and positive. We did not show statistically significant differences in estrogen β expression between melanoma and nevi melanocytes. We have shown statistically significant differences in estrogen β expression in margin melanocytes between common and dysplastic nevi. The expression of estrogen receptor β was higher in margin of dysplastic nevi (median 90% vs. 100%, *p* = 0.046). Keratinocytes of melanomas and dysplastic nevi showed differences in estrogen β receptor-expression was lower in melanoma tissue (median 80% vs. 90%, *p* = 0.021). Those differences may potentially show the impact of estrogen receptor β expression on microenvironment surrounding the tumor or may be secondary to genetic predispositions of individuals.

In accordance with previous reports we showed that melanomas are characterized by high expression of estrogen receptor β and low expression of estrogen receptor α. Nading et al. examined the ER α and ERβ expression in 17 common nevi, 11 dysplastic nevi and 5 congenital nevi in pregnant individuals and compared them with 62 dysplastic nevi and 35 congenital nevi from non-pregnant patients, and found ERα was expressed solely in sebocytes of sebaceous glands, while ERβ expression was elevated in nevi, especially acquired nevi [38]. Zhou et al. evaluated the expression of estrogen α and β receptors in 18 pregnant women, women up to 6 months following the labor, 18 non-pregnant women and 18 men diagnosed with melanoma. Estrogen receptor α expression was shown in 2 cases of acral lentiginous melanomas—in one female with melanoma diagnosed during pregnancy and in one male. Twenty-two cases expressed estrogen receptor β: 10 samples from pregnant patients (56%), 7 from non-pregnant individuals (39%) and 5 from men (29%) from control group [33]. Ohata et al. assessed the expression of estrogen receptor α receptor in 40 melanocytic lesions (primary melanomas and nevi). Melanocytic nevi did not express estrogen receptor α. The study showed estrogen β expression in 100% of nevus cells of each assessed lesion. All melanoma melanocytes expressed estrogen receptor β, they did not express estrogen receptor α. There were no differences in cell staining intensity between women, men and pregnant women [39]. In our study we used different criterion of assessment of melanocytic nevi. We evaluated the percentage of cells stained in 1 mm^2^.

GPER is a protein which is activated by and binds to estradiol and mediates rapid and delayed transcription. GPER activation is connected with transactivation of epidermal growth factor, mitogen-activated kinase, ERK kinase, and 3-phosphatydyloinositole pathways, which are involved in pathogenesis of melanoma [40,41,42,43]. GPER regulates transcription factors, their co-activators, as well as cell cycle proteins (cyclin D2) and programmed cell death proteins (Bcl-2) [44,45]. In mouse melanoma cells, GPER and GPER agonist G-1 decreased cell divisions by inhibiting cell cycle in G2 phase, reduced the level of phosphorylated ERK 1/2 similarly to the antiestrogen tamoxifen [46]. Natale et al. exposed melanoma cell lines to estrogen and GPER agonist G-1 and showed dose-dependent inhibition of melanoma cells proliferation possibly by inhibition of c-Myc protein and protein kinase A [47].

We found that GPER expression was higher in nuclei and cytoplasm of dysplastic nevi and at the margin compared to melanoma as well as in sebaceous glands of tissue surrounding dysplastic versus common nevi. GPER expression was significantly higher in skin margin tissue melanocytes compared to melanoma melanocytes while there were no differences in keratinocytes of melanoma margin and melanoma tissue. Similar nuclear expression rates were shown in non-pregnancy related melanoma in a study of 38 pregnancy-associated melanomas and 43 non-pregnancy-associated melanomas [47]. This study also found that melanomas co-expressing GPER/ER β were more common in pregnant women compared to non-pregnant individuals. Finally, GPER/ERβ co-expression was associated with lower Breslow thickness, mitotic rate and higher peritumoral lymphocyte infiltration which are associated with improved disease-free survival [45]. We did not find any previous studies comparing the expression of estrogen receptors α, β and GPER between common nevi, dysplastic nevi and melanomas.

The difference in ERβ and GPER expression in margins and sebaceous glands in melanoma and non-melanoma lesions suggest their potential role as diagnostic biomarkers. However future studies should further evaluate GPER and ERβ expression in melanoma, particularly in high-estrogen states such as pregnancy and evaluate if they could be used as prognostic factors.

## Figures and Tables

**Table 1:** Comparison of receptor expression between common nevi, dysplastic nevi and melanomas.

Receptor Expression	*p*-Value	Comparison of Common Nevi and Dysplastic Nevi—*p*-Value	Comparison of Common Nevi and Melanomas—*p*-Value	Comparison of Dysplastic Nevi and Melanomas—*p*-Value
ERα in keratinocytes of margin	0.22	0.99	0.37	0.49
ERα in keratinocytes of melanocytic lesion	0.21	0.99	0.41	0.39
ERα in melanocytes of margin	0.31	0.99	0.57	0.84
ERα in melanocytes of melanocytic lesion	0.91	0.99	0.99	0.99
ERα in sweat glands	0.15	0.99	0.18	0.78
ERα in sebaceous glands	0.19	0.99	0.28	0.56
ERβ in keratinocytes of margin	0.48	0.78	0.99	0.99
ERβ in keratinocytes of melanocytic lesion	0.02 *	0.39	0.67	0.02 *
ERβ in melanocytes of margin	0.04 *	0.05 *	0.67	0.71
ERβ in melanocytes of melanocytic lesion	0.58	0.99	0.99	0.99
ERβ in sweat glands	0.71	0.99	0.99	0.99
ERβ in sebaceous glands	0.64	0.99	0.99	0.99
GPER in keratinocytes of margin	0.82	0.99	0.99	0.99
GPER in keratinocytes of melanocytic lesion	0.09	0.23	0.99	0.14
GPER in melanocytes of margin	0.00 *	0.99	0.00 *	0.02 *
GPER in melanocytes of melanocytic lesion cytoplasm	0.02 *	0.99	0.08	0.02 *
GPER in melanocytes of melanocytic lesion nucleus	0.04 *	0.58	0.65	0.04 *
GPER in sweat glands	0.01 *	0.03 *	0.06	0.99
GPER in sebaceous glands	0.03 *	0.03 *	0.26	0.99

ER: Estrogen receptor, GPER: G-protein coupled estrogen receptor. *p*-value for Kruskal-Wallis ANOVA test for differences in hormonal receptors expression between common nevi, dysplastic nevi and melanomas, and for post-hoc analysis of Kruskal-Wallis test. * statistically significant for *p* < 0.05.

**Table 2:** Estrogen α, β and GPER expression in melanocytic lesions and surgical margin-descriptive statistics.

	Common Nevi			Dysplastic Nevi			Melanomas		
Receptors Expression	No	Mean (SD)	Median (IQR)	No	Mean (SD)	Median (IQR)	No	Mean (SD)	Median (IQR)
ERα in keratinocytes of margin	25.00	12.00 (14.36)	5 (5.00–15.00)	24.00	9.83 (9.57)	7.50 (3.00–15.00)	24.00	8.13 (12.58)	5.00 (0.00–10.00)
ERα in lesion keratinocytes	25.00	11.60 (12.39)	10.00 (5.00–15.00)	24.00	10.88 (9.70)	10.00 (5.00–17.50)	24.00	10.00 (16.08)	5.00 (0.00–12.50)
ERα in melanocytes of margin	25.00	42.20 (49.16)	0.00 (0.00–100.00)	24.00	37.29 (44.79)	2.50 (0.00–90.00)	24.00	16.46 (30.38)	0.00 (0.00–15.00)
ERα in lesion melanocytes	25.00	16.20 (18.72)	10.00 (5.00–20.00)	24.00	17.54 (20.07)	10.00 (5.00–30.00)	24.00	20.63 (26.18)	5.00 (2.50–35.00)
ERα in sweat glands	21.00	8.57 (12.56)	5.00 (0.00–10.00)	24.00	12.71 (19.50)	5.00 (0.00–17.50)	24.00	17.50 (19.95)	10.00 (5.00–30.00)
ERα in sebaceous glands	21.00	8.57 (11.20)	5.00 (0.00–10.00)	24.00	11.67 (16.80)	5.00 (0.00–17.50)	24.00	17.50 (19.95)	10.00 (5.00–30.00)
ERβ in keratinocytes of margin	25.00	80.40 (16.95)	90.00 (80.00–90.00)	24.00	85.63 (13.30)	90.00 (80.00–90.00)	24.00	78.33 (24.96)	90.00 (70.00–90.00)
ERβ in lesion keratinocytes	25.00	83.60 (13.50)	90.00 (80.00–90.00)	24.00	88.13 (11.87)	90.00 (90.00–92.50)	24.00	75.21 (23.38)	80.00 (65.00–90.00)
ERβ in melanocytes of margin	25.00	82.80 (26.06)	90.00 (80.00–90.00)	24.00	90.00 (22.60)	100.00 (90.00–100.00)	24.00	82.71 (32.40)	90.00 (90.00–100.00)
ERβ in lesion melanocytes	25.00	81.60 (21.15)	90.00 (80.00–90.00)	24.00	78.75 (23.74)	90.00 (70.00–92.50)	24.00	68.13 (33.65)	85.00 (50.00–90.00)
ERβ in sweat glands	22.00	84.55 (17.66)	90.00 (90.00–90.00)	24.00	87.29 (13.75)	90.00 (80.00–100.00)	23.00	83.26 (21.72)	90.00 (80.00–95.00)
ERβ in sebaceous glands	19.00	83.68 (18.92)	90.00 (80.00–90.00)	24.00	87.29 (13.75)	90.00 (80.00–100.00)	21.00	82.14 (22.39)	90.00 (80.00–90.00)
GPER in keratinocytes of margin	25.00	38.00 (24.79)	40.00 (10.00–50.00)	24.00	40.62 (27.12)	35.00 (20.00–70.00)	24.00	36.67 (28.96)	40.00 (10.00–60.00)
GPER in lesion keratinocytes	25.00	39.60 (24.28)	40.00 (20.00–60.00)	24.00	53.96 (28.32)	60.00 (40.00–75.00)	24.00	37.29 (29.12)	30.00 (10.00–65.00)
GPER in melanocytes of margin	25.00	86.40 (32.90)	100.00 (100.00–100.00)	24.00	85.42 (33.49)	100.00 (95.00–100.00)	24.00	55.00 (44.92)	80.00 (0.00–100.00)
GPER in lesion melanocytes-cytoplasm	25.00	53.60 (20.39)	50.00 (40.00–70.00)	24.00	57.50 (24.00)	70.00 (40.00–80.00)	24.00	36.67 (28.23)	30.00 (15.00–60.00)
GPER in lesion melanocytes-nucleus	25.00	39.80 (25.76)	40.00 (20.00–60.00)	24.00	50.63 (28.18)	45.00 (30.00–75.00)	24.00	30.21 (25.47)	25.00 (10.00–40.00)
GPER in sweat glands	24.00	41.25 (30.08)	50.00 (10.00–70.00)	24.00	64.79 (26.02)	70.00 (50.00–80.00)	23.00	62.17 (26.79)	70.00 (50.00–90.00)
GPER in sebaceous glands	24.00	41.67 (29.70)	50.00 (10.00–70.00)	24.00	64.79 (26.02)	70.00 (50.00–80.00)	23.00	57.83 (28.44)	70.00 (40.00–70.00)

ER: Estrogen receptor, GPER: G-protein coupled estrogen receptor. Percentage of receptor expression: estrogen receptor α, beta, and GPER in common nevi, dysplastic nevi, skin melanomas, and healthy skin margins. IQR–interquartile range, No-number of lesions.

**Table 3:** The comparison of expression of estrogen receptors (α, β and GPER) between melanocytes and keratinocytes of melanomas and healthy tissue margin.

Pair of Variables	*p* Value	Median Expression in Melanoma Tissue	Comparison of Common Nevi and Melanomas—*p*-Value	Median Expression in Margin Tissue	IQR-Expression in Margin Tissue
Estrogen receptor α expression in healthy skin margin keratinocytes vs. melanoma keratinocytes	0.11	5.00	0.00–12.50	5.00	0.00–10.00
Estrogen receptor α expression in healthy skin margin melanocytes vs. melanoma melanocytes	0.24	5.00	2.50–35.00	0.00	0.00–15.00
Estrogen receptor β expression in healthy skin margin keratinocytes vs. melanoma keratinocytes	0.20	80.00	65.00–90.00	90.00	70.00–90.00
Estrogen receptor β expression in healthy skin margin melanocytes vs. melanoma melanocytes	0.01 *	85.00	50.00–90.00	90.00	90.00–100.00
GPER receptor expression in healthy skin margin keratinocytes vs. melanoma keratinocytes	0.84	30.00	10.00–65.00	40.00	10.00–60.00
GPER receptor expression in healthy skin margin melanocytes vs. melanoma melanocytes	0.02 * 0.03 *	25.00 nuclear; 30.00 cytoplasmic	10.00–40.00 nuclear; 15.00–60.00 cytoplasmic	80.00 nuclear and cytoplasmic	0.00–100.00 nuclear nad cytoplasmic

ER: Estrogen receptor, GPER: G-protein coupled estrogen receptor. * statistically significant for *p* < 0.05.

## Data Availability

Data are available upon reasonable request to the Corresponding author.
